# Supplementary material for: Biosynthesis of silver nanoparticles using Caesalpinia ferrea (Tul.) Martius extract: physicochemical characterization, antifungal activity and cytotoxicity
Source: PeerJ. 2018 Mar 19;6:e4361. doi: 10.7717/peerj.4361 (PMC5863706; doi:10.7717/peerj.4361)
Supplement: Supplemental Information 1 [file peerj-06-4361-s001.pptx]

## Slide 1
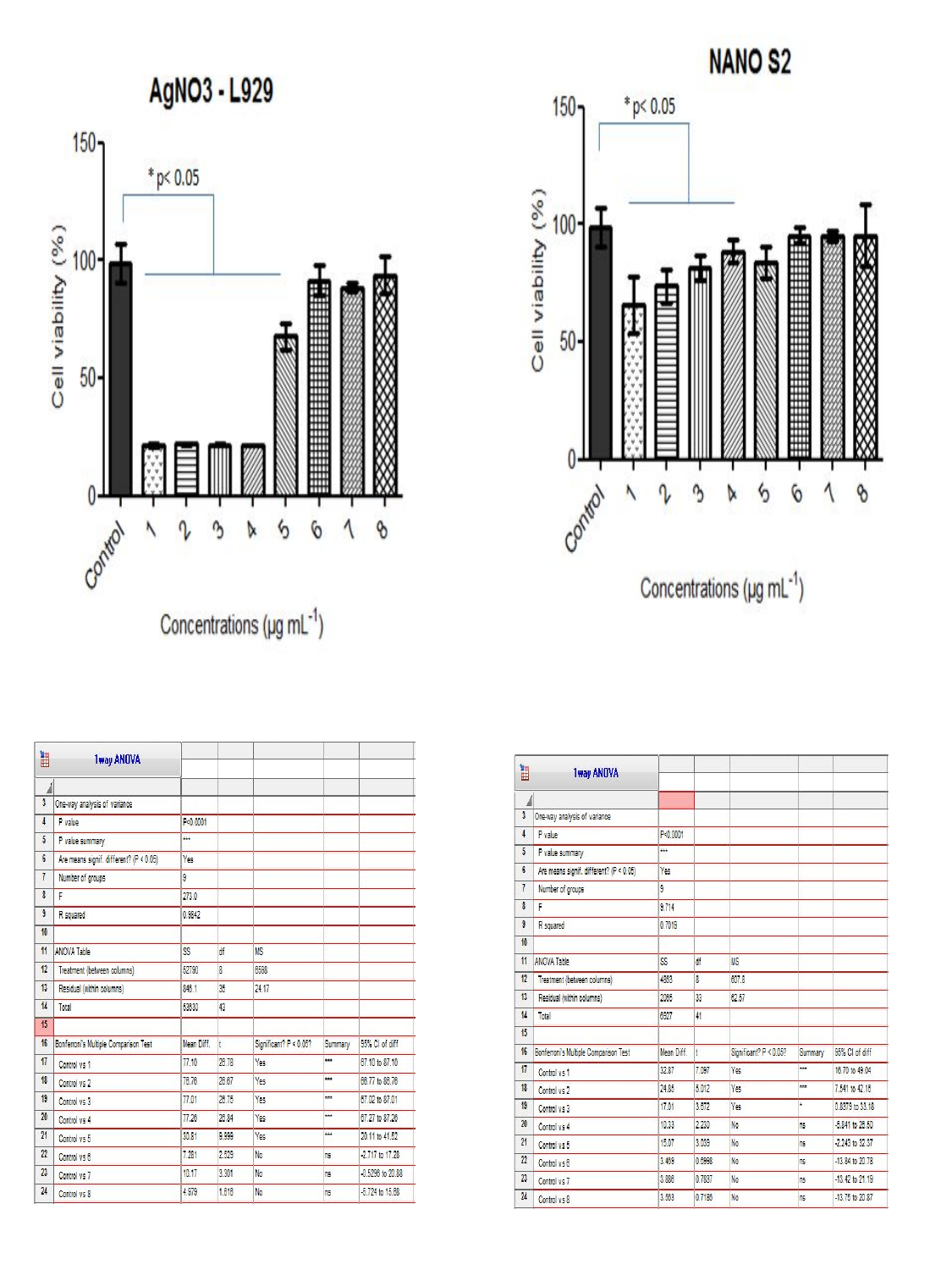

## Slide 2
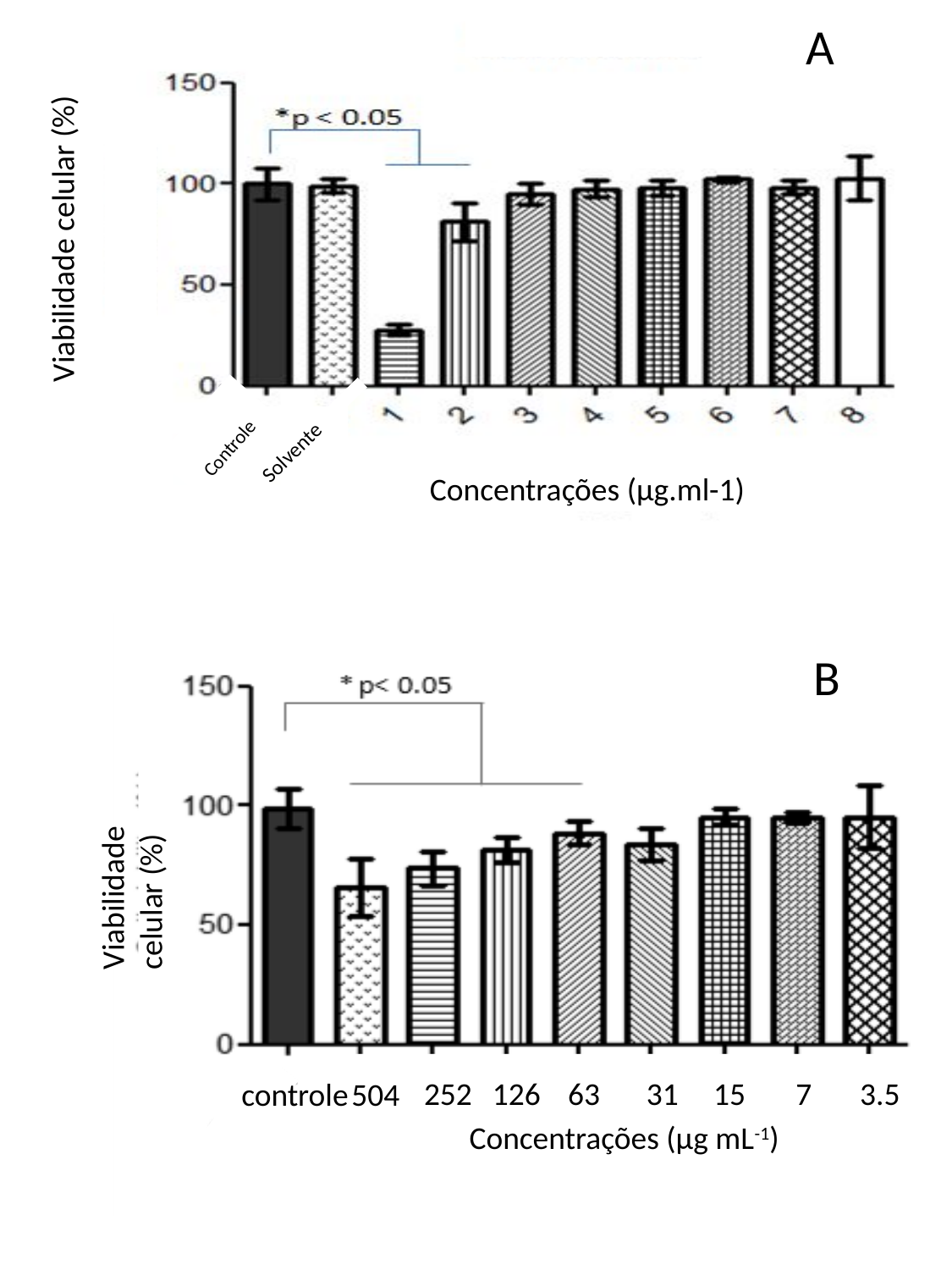

A
Viabilidade celular (%)
Controle
Solvente
Concentrações (µg.ml-1)
B
Viabilidade celular (%)
252
126
63
31
15
7
3.5
504
controle
Concentrações (µg mL-1)

## Slide 3
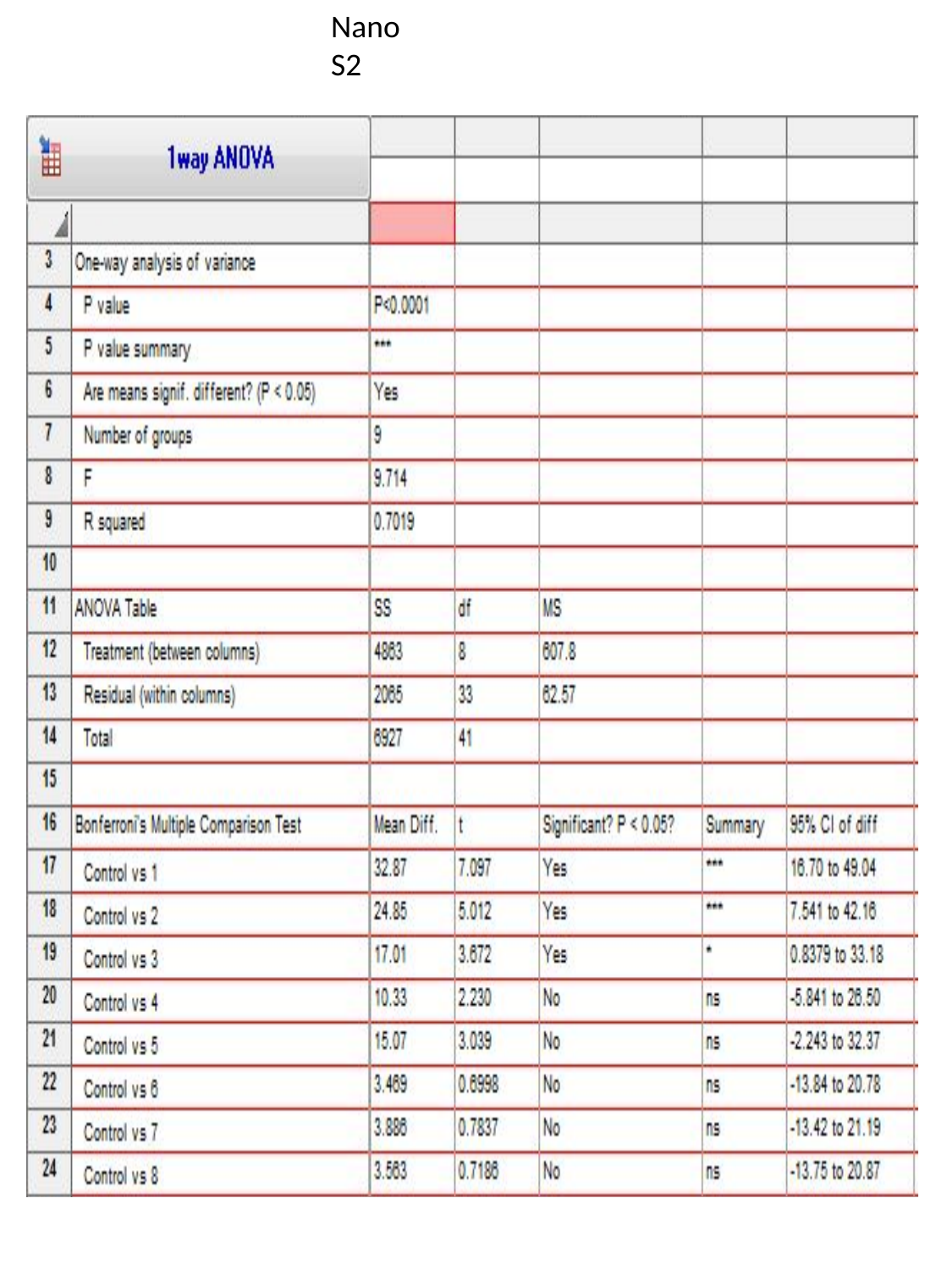

Nano S2

## Slide 4
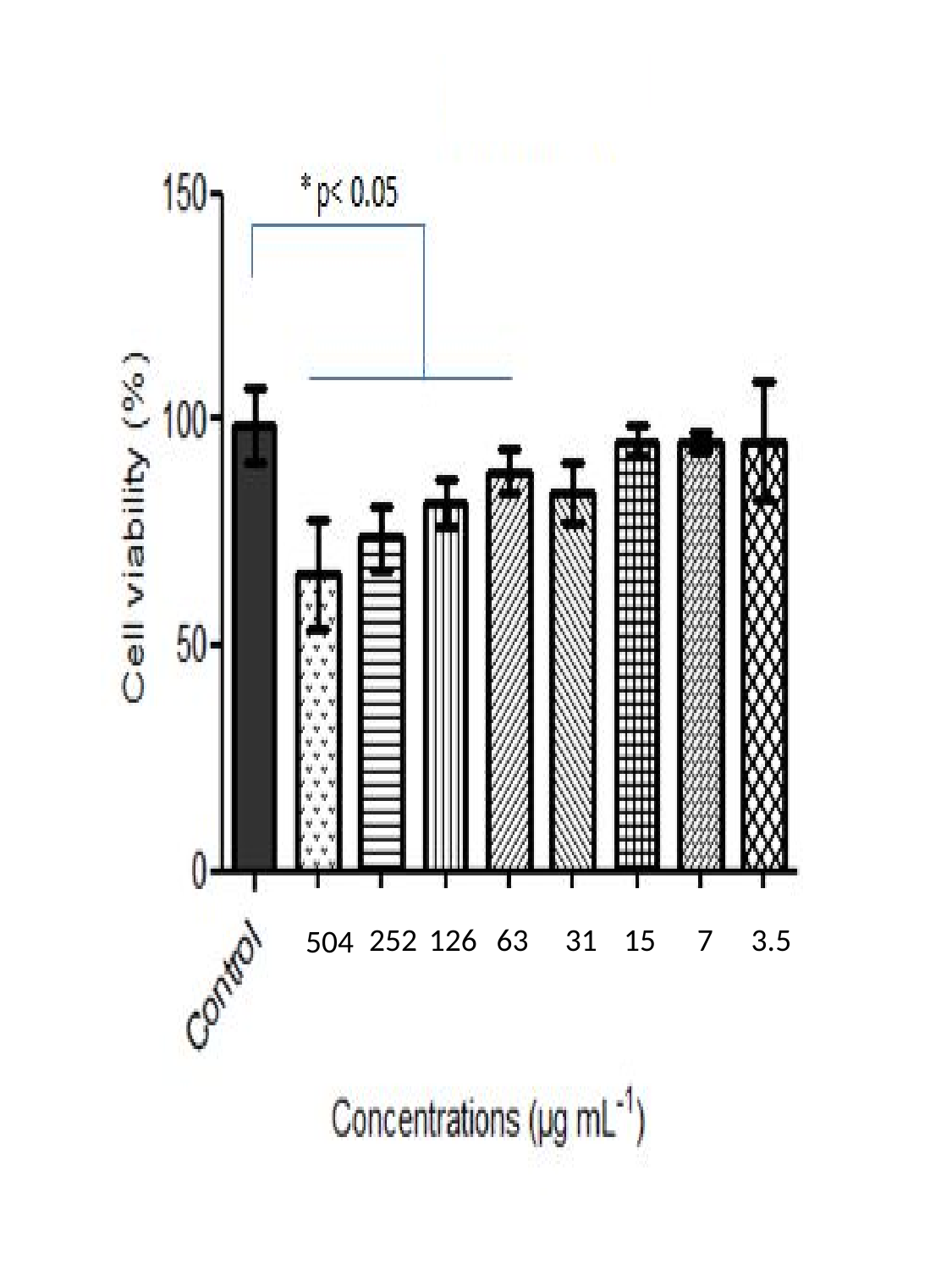

252
126
63
31
15
7
3.5
504

## Slide 5
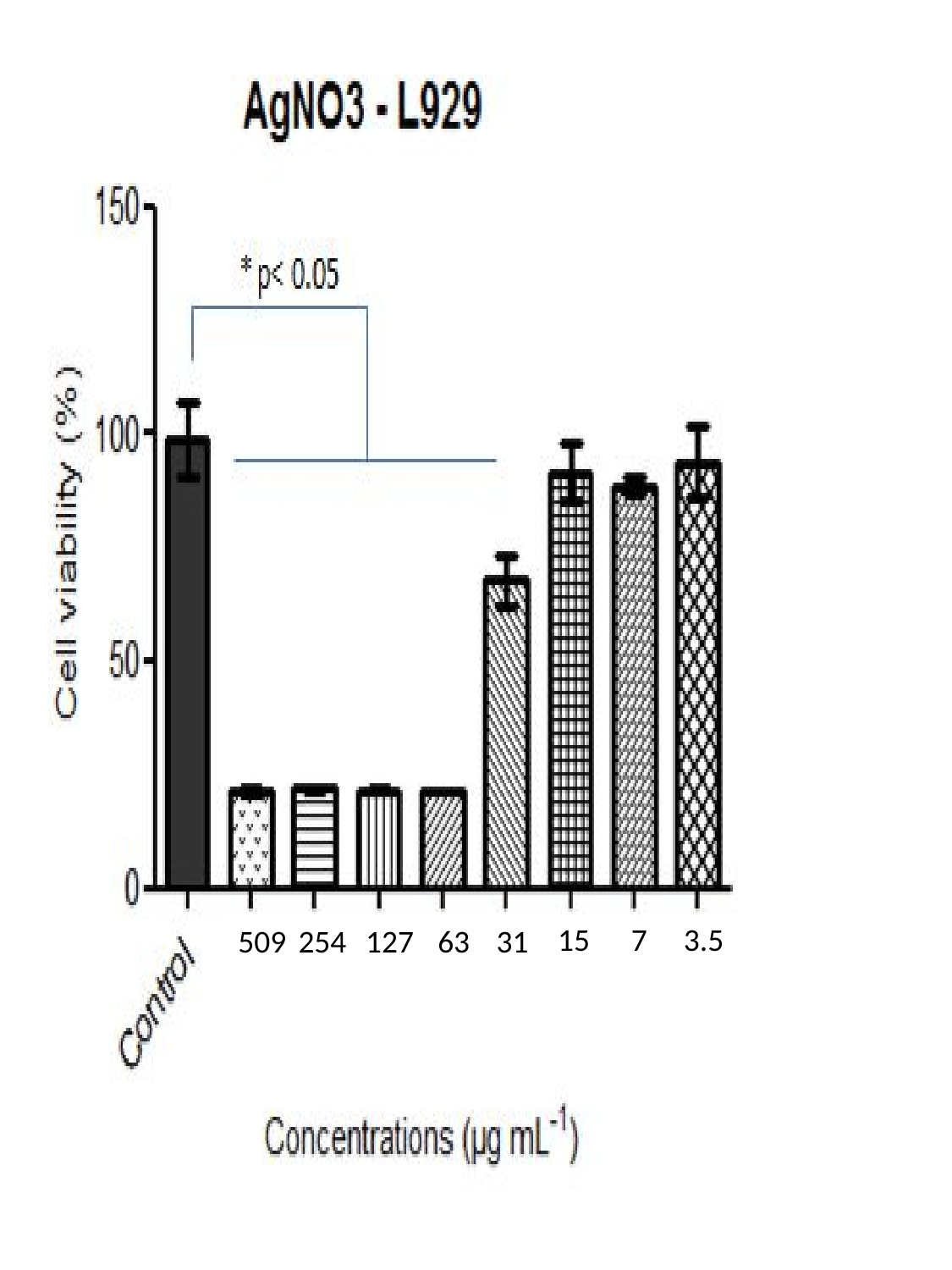

15
7
3.5
509
254
127
63
31

## Slide 6
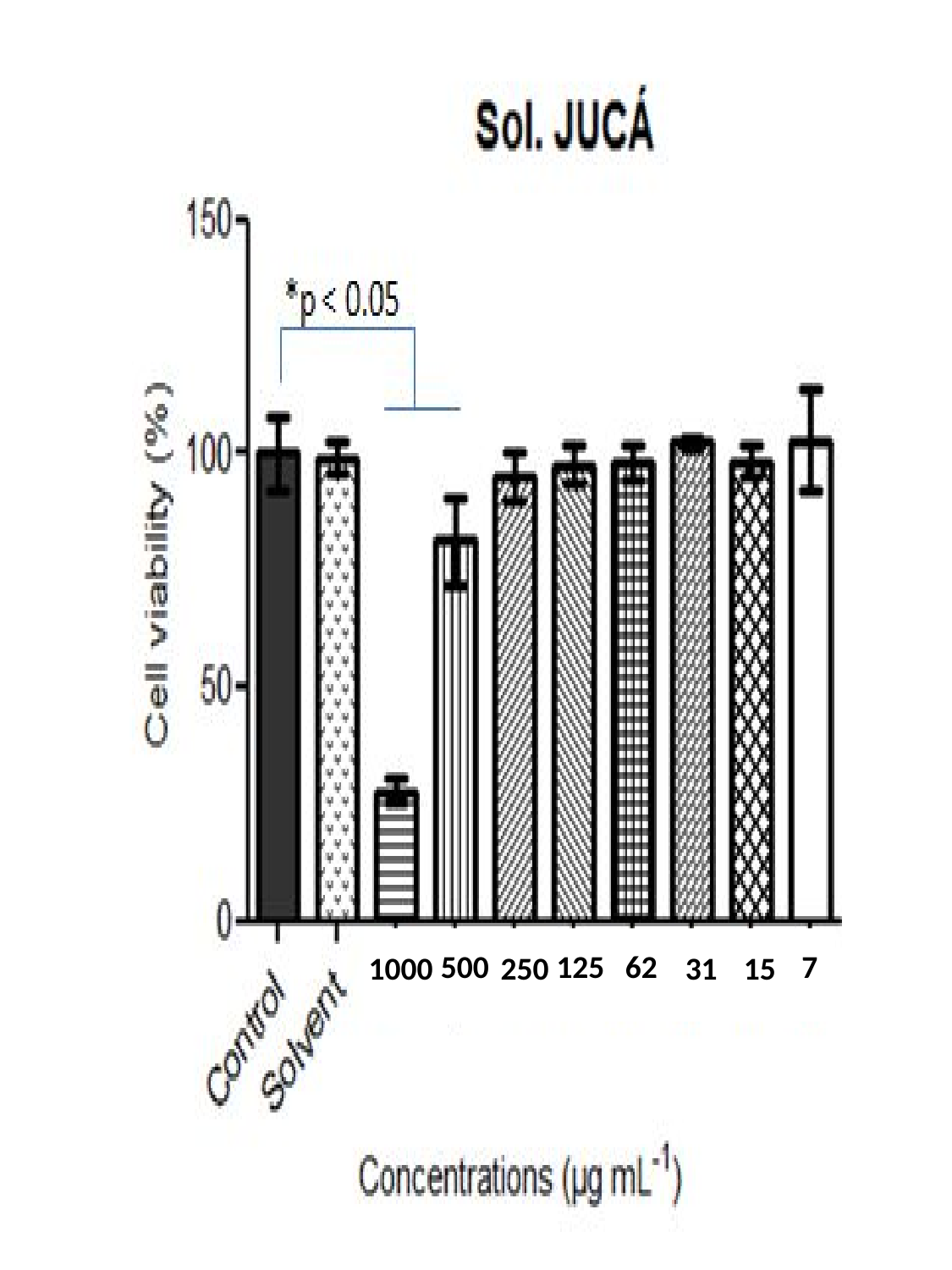

500
125
62
7
1000
250
31
15
